# Advances of Naturally Derived and Synthetic Hydrogels for Intervertebral Disk Regeneration

**DOI:** 10.3389/fbioe.2020.00745

**Published:** 2020-06-30

**Authors:** Guoke Tang, Bingyan Zhou, Feng Li, Weiheng Wang, Yi Liu, Xing Wang, Chao Liu, Xiaojian Ye

**Affiliations:** ^1^Department of Orthopedic Surgery, Changzheng Hospital, Second Military Medical University, Shanghai, China; ^2^Department of Spine Surgery, The Affiliated Zhuzhou Hospital of Xiangya School of Medical CSU, Zhuzhou, China; ^3^Beijing National Laboratory for Molecular Sciences, Institute of Chemistry, Chinese Academy of Sciences, Beijing, China; ^4^University of Chinese Academy of Sciences, Beijing, China

**Keywords:** intervertebral disk regeneration, natural hydrogel, synthetic hydrogel, low back pain, tissue engineering

## Abstract

Intervertebral disk (IVD) degeneration is associated with most cases of cervical and lumbar spine pathologies, amongst which chronic low back pain has become the primary cause for loss of quality-adjusted life years. Biomaterials science and tissue engineering have made significant progress in the replacement, repair and regeneration of IVD tissue, wherein hydrogel has been recognized as an ideal biomaterial to promote IVD regeneration in recent years. Aspects such as ease of use, mechanical properties, regenerative capacity, and their applicability as carriers for regenerative and anti-degenerative factors determine their suitability for IVD regeneration. This current review provides an overview of naturally derived and synthetic hydrogels that are related to their clinical applications for IVD regeneration. Although each type has its own unique advantages, it rarely becomes a standard product in truly clinical practice, and a more rational design is proposed for future use of biomaterials for IVD regeneration. This review aims to provide a starting point and inspiration for future research work on development of novel biomaterials and biotechnology.

## Introduction

The vertebral column is composed of the rigid bony vertebral body, interspersed with intervertebral disks (IVDs) and facet joints (Twomey and Taylor, 1985). It protects our spinal cord and supports our head, upper extremities and torso while providing flexibility in multiple degrees of freedom. The IVD accounts for one-third of the spinal column in total by height, enabling the movement of spinal column and transfer the loads associated with movement (Risbud and Shapiro, 2014). Owing to the limited repair capacity of non-vascular and non-synovial structures, the IVDs have been shown to be prone to cumulative damage. Chronic low back pain is closely related to the degeneration of the lumbar vertebrae IVD, while root pain is connected with the protrusion of posterior annulus fibrosus (AF) and nucleus pulposus (NP) (Vos et al., 2012). IVD degeneration is a chronic disease that can slowly reduce the content of IVD, causing the instability and thereby limiting the mobility of spinal cord. Low back pain caused by IVD degeneration can start early in life (before the age of 20) (Lewin, 1955; Miller et al., 1988), and at least 60% of people over the age of 70 will be affected, which is the most common reason for disability in developed countries (Risbud and Shapiro, 2014). According to the stage of degenerative changes, current clinical treatment strategies are generally classified into conservative and surgical treatments. However, on account of the incomplete understanding of pathological biology of IVD degeneration, these strategies are limited to alleviating pain and symptoms instead of the elimination of disease itself (Wu et al., 2006; Huang et al., 2014; Wang F. et al., 2015; Wang K. et al., 2015; Wang et al., 2016a).

**FIGURE 1:**
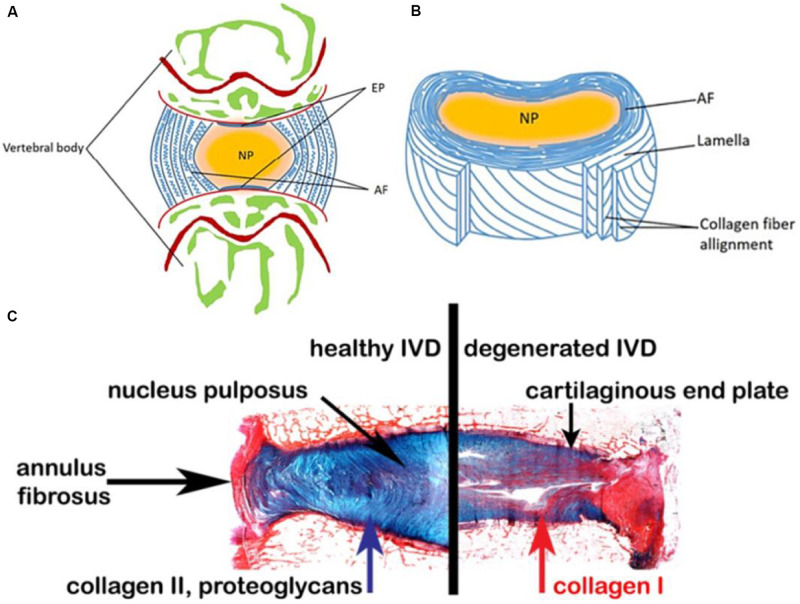
Schematic representations of the adult IVD. **(A)** Midsagittal cross-section showing anatomical regions. **(B)** Three-dimensional view of the AF lamellar structure. **(C)** Comparison of a healthy human disk and a degenerated disk. Reproduced from Sakai and Andersson (2015); Eyre and Muir (1976) with permissions from Copyright 2015 AO Research Institute and 2019 MDPI.

The main function of IVD is to transmit mechanical force to the spine and maintain an active segment for the bending, stretching and rotation movements (Sakai and Andersson, 2015). From an anatomic point of view, a healthy IVD can be roughly divided into three regions (Figure 1): (1) NP, a gel-like core, consists of type II collagen and proteoglycans, and the NP cells are highly hydrated for the strength and mobility support of spine (Eyre and Muir, 1976; Sowa et al., 2008; Pereira et al., 2013; Schutgens et al., 2015); (2) AF, a multi-layered fibrous tissue around the NP, is organized with the components of stacked lamellae predominantly of type I collagen. AF needs to transfer the stress from NP to maintain the IVD integrity and protect it from bending, stretching and torsion injury (Yu et al., 2005; Schollum et al., 2008; Shankar et al., 2009; Isaacs et al., 2014; Nakamichi et al., 2016); (3) Endplates (EPs) are two pieces of hyaline cartilage wrapped by upper and lower vertebral bodies at their junctions, and their micropores play an important role in transporting nutrients into the disk (Roberts et al., 1989).

So far, traditional treatments of lower back pain mainly include physiotherapy, analgesia, muscle relaxants, corticosteroids, and surgery (Jacobs et al., 2013). Although spinal fusion may reduce pain, surgical treatment is highly invasive and not benefited for biological repair and preservation of motion of the treated segment (Radcliff et al., 2013). In terms of arthroplasty, there are currently no long-term effective and completely safe IVD substitutes (Thavaneswaran and Vandepeer, 2014). Although discectomy and fusion are the two frequently used surgical approaches for treating IVD degeneration, they are not ideal treatments due to the alteration of biomechanics for the spine (Goel et al., 1986; Hanley and Shapiro, 1989; Bao et al., 1996; Gloria et al., 2007). Therefore, an artificial disk is recognized as an effective method for the degenerated disk (Bao and Yuan, 2000; Gloria et al., 2011), but it is difficult to design ideal IVD prostheses for reproducing the native structures with required durability on account of the complex structure and function of IVD. It has been well reported that currently available IVD prostheses on the market are basically unqualified due to the abrasion or mismatch between natural tissues and biomechanical properties (Shikinami et al., 2004). To stimulate IVD regeneration, an ideally biological tissue replacement should possess high strength, flexibility and toughness, but a single structural arrangement of biomaterials does not allow combination of all these features. Accordingly, a biomimetic approach has been adopted to reproduce the structure of natural disk to overcome the drawbacks of current prostheses (Kalson et al., 2008).

Hydrogels, possessing 3D cross-linked network structures, tunable physicochemical properties and similar structures of extracellular matrix (ECM) for the cell adhesion and proliferations, have attracted a great deal of attentions in IVD regeneration (Shu et al., 2006; Wang et al., 2017a, 2018a, 2020; Bao et al., 2019, 2020; Zhu et al., 2019; Fan et al., 2020; Li et al., 2020a; Liu H. Y. et al., 2020). The hydrogel designs contemplate the implant performance in terms of structural integrity, biocompatibility, biodegradability, safety, solute transport of cellular behavior, mechanical strength, and low viscosity. In this respect, hydrogels are versatile by varying material types, molecular weight, crosslinking degree, chemical surface, solid contents and functionalization, thus they could be utilized to mimic the mechanical properties of native tissues (Yang et al., 2016, 2018; Cao et al., 2018). Most of hydrogels can be implanted *in vivo* with minimal invasive techniques. The standard approach consists of a three-dimensional (3D) biomaterial scaffold with gene vectors, soluble factors and/or biochemical signals to promote the new tissue generation (Li et al., 2020b; Liu Z. Y. et al., 2020; Xu et al., 2020). Furthermore, their crosslinked architectures may provide tissue-like viscoelastic, diffusive transport and interstitial flow characteristics. Hydrogels are often divided into naturally derived hydrogels and synthetic hydrogels that have been used to exploit the regenerative capacities of host tissues or transplanted cells (Yu et al., 2020). Naturally derived hydrogels are particularly appealing because of their inherent biocompatibility, biodegradability and safety, including chitosan, alginate, hyaluronan, collagen and agarose, which are generally obtained from various renewable resources like animal, plant, algae, and microorganisms in the great world (Mano et al., 2007). Synthetic hydrogels possess tunable properties for facile fabrication of functional productions, which mainly contain polyethylene glycol (PEG), polycarbonate urethane (PU), and poly(epsilon-caprolactone) (PCL). However, it’s necessary to ensure that contaminants, unreacted reagents, surplus monomers, catalysts and other byproducts are completely removed to ensure the biosafety of synthetic hydrogels.

The focus of this review is to describe the state of the art of different biological hydrogels and hydrogel scaffolds for IVD regeneration, and discuss their advantages and drawbacks with a focus on biodegradable tissue replacement of the NP and AF for IVD regeneration.

## Naturally Derived Hydrogels

### Chitosan

Chitosan is a kind of biological polysaccharide with the components of glucosamine and N-acetylglucosamine, which has been widely applied in wound hemostasis, anti-infection, drug delivery, and gene delivery (Di Martino et al., 2005). Chitosan is degradable *in vivo* through lysozyme activity, and increase of the deacetylation degree can prolong its degradation time. Although the cationic nature of chitosan facilitates their interactions with anionic glycosaminoglycans *in vivo* and combination of growth factors for improving the loading capacity, chitosan hydrogel is softer than native NP (Sasson et al., 2012). In general, chitosan hydrogels were added with other biological materials (e.g., alginate and gelatin) to improve the biomechanical properties (Cheng et al., 2010). For example, implanting the chitosan/dextran hydrogel into the spine of animal body displayed no squeeze and extrusion under a large range of loads, and Young’s modulus and Poisson’s ratio were similar to native IVDs under the unconfined compression (Smith et al., 2014).

**FIGURE 2:**
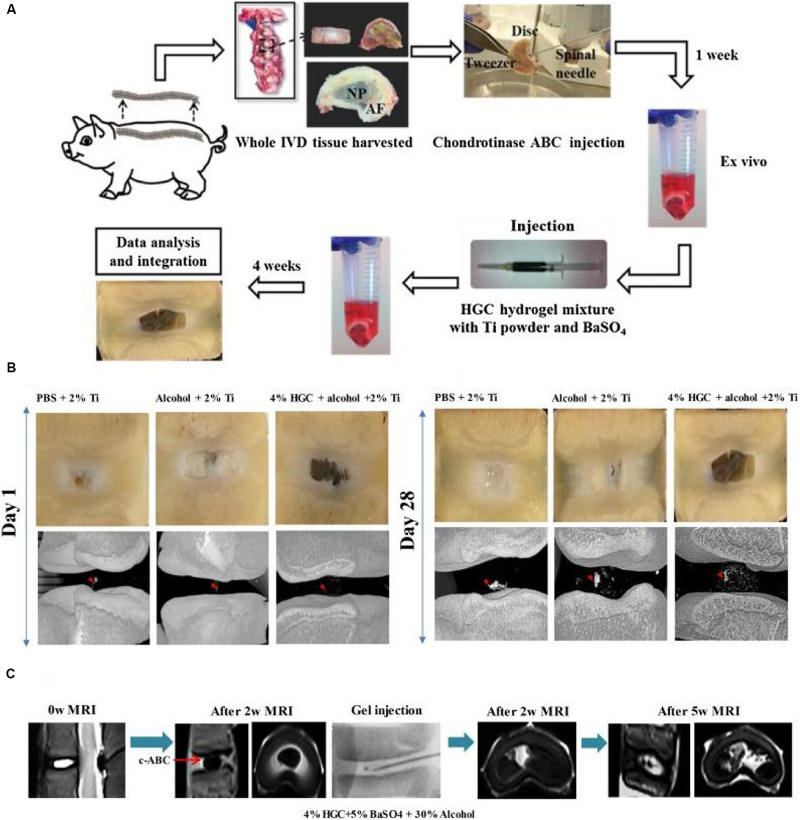
**(A)** Schematic description of the evaluation of *ex vivo* degenerative IVD tissue and treatment with HGC hydrogel for CT and MRI imaging. **(B)** Macroscopic observation and micro-CT images of degenerated IVD tissue at 1 day and 28 days after injection with hydrogels. **(C)** MRI images of IVD tissue before and 2 weeks after the disk degeneration process and subsequent degenerative IVD tissue at 2 weeks and 5 weeks after injection of HGC hydrogel in 30% alcohol solution. Reproduced from Li et al. (2018) with permission from Copyright 2018 Elsevier.

Alinejad et al. (2019) prepared a kind of thermosensitive chitosan hydrogels via combinations of chitosan with sodium hydrogen carbonate and/or β-glycerophosphate in PBS solutions. They possessed higher mechanical properties of compression and torsion than human NP tissue. Hydrogel cytocompatibility and functionality were assessed by measuring cell viability, metabolism and proteoglycan synthesis. The usage of injectable formulations was suitable for IVD treatment to reduce low back pain and maintain the biomechanical multifunction (Alinejad et al., 2019). Li et al. developed a thermo-sensitive injectable hydrogel from N-hexanoylation of glycol chitosan to promote IVD regeneration (Figure 2). Depending on the hexanoylation degree and polymer concentration, these hydrogels expressed a sol-gel transition from 23 to 56°C. After implanting the hydrogels into the defective IVD using a porcine model, no cytotoxicity and adverse effects were found within 4 weeks *in vivo*, which indicated their alternative effects for the treatment of disk herniation (Li et al., 2018).

It should be noted that these versatile chitosan materials are frequently used with other components to improve their mechanical properties. Although chitosan hydrogels can promote the cartilage differentiation, their poor solubility in neutral conditions and harsh processability greatly restrict the range of applications.

### Alginate

Alginate, a natural biopolymer extracted from the brown algae (Bron et al., 2011), can crosslink via ionic, covalent and thermal processes to form the alginate hydrogels, which have been applied in drug release and wound dressing fields. The stiffness of alginate hydrogel is adjusted by changing the weight/volume (w/v) percentage of alginate. When the 2% (w/v) of alginate hydrogel was immersing into CaCl_2_ solutions, the generated alginate hydrogel can achieve the matched requirement of native IVD (Foss et al., 2014). In addition, alginate hydrogels are usually combined with synthetic polymers such as PEG or PCL or added to naturally derived hydrogels to form the physical cross-linking networks to improve their mechanical properties.

**FIGURE 3:**
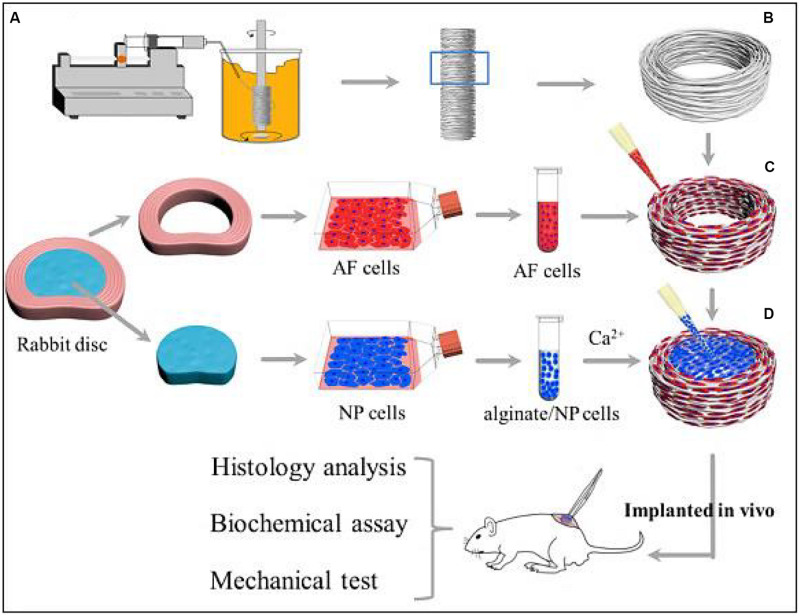
Schematic illustration shows the construction of composite TE-IVD and the design of *in vitro* study and *in vivo* implantation into nude mice. **(A)** Wet spinning process. **(B)** Circumferentially oriented PCL AF scaffold. **(C)** Seeding cells to the AF phase. **(D)** Injecting alginate containing cells into the NP phase. Reproduced from Du et al. (2019) with permission from Copyright 2019 Elsevier.

More importantly, the crosslinking alginate hydrogels can improve the *in vivo* synthesis of ECM and facilitate the adhesion, growth and proliferation of AF, NP, and EPs-derived stem cells. Compared to AF and NP-derived cells, EPs-derived cells had the strongest regenerative ability and produced preferable NP regeneration in rabbit models of disk degeneration. Adding glucosamine and chondroitin sulfate into alginate hydrogel can effectively promote NP cell differentiation and matrix production (Melrose et al., 2001; Wang et al., 2014). Du et al. (2019) proposed a biomimetic AF-NP composite with oriented PCL microfibers seeded with AF cells and with NP cells-loaded alginate hydrogel as a core. Similar to the native IVD, AF cells could spread along the circumferentially oriented PCL microfibers while NP cells colonized in the alginate hydrogels without interpenetrating between AF and NP areas (Figure 3). This engineered IVD was subcutaneously implanted into nude mice and expressed the favorable IVD-like tissue formation (Du et al., 2019).

In short, although alginate hydrogel is a versatile biomaterial with promising prospects as a scaffold for NP regeneration, the main drawback of this material is that the mechanism of degradation *in vivo* is not fully understood yet.

### Hyaluronan

**FIGURE 4:**
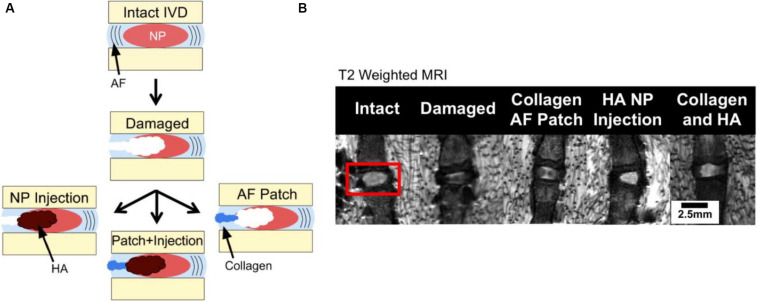
**(A)** Segments test via annulotomy/nucleotomy and **(B)** MRI images of NP hydration and morphology where hyperintense regions correspond with greater relaxation time and hydration. Reproduced from Sloan et al. (2017) with permission from Copyright 2017 Elsevier.

Hyaluronic acid (HA) comprising of repeating units of D-glucuronic acid and N-acetyl-D-glucosamine residues is a connective tissue polysaccharide for tissue regeneration, drug delivery, and wound healing applications (Kenne et al., 2013). Adjustment of the percentage of HA can enhance the stiffness of native NP due to the reservation of rounded morphology of NP cells with a high viability and activity, and promote the ECM production for IVD regeneration. Therefore, HA hydrogels in a clinical setting possess many advantages on the possibility of implanted biomaterials with the minimally invasive techniques (Chung et al., 2009). Kazezian et al. (2017) prepared a HA gel with anti-inflammatory and matrix modulatory effect on down-regulates IFNARI, IFNAR2, STAT1/2, JAK1, IFIT3, and IGFBP3 mRNA expression in the inflamed groups. In the ECM, aggrecan and collagen I were up-regulated and ADAMTS4 was down-regulated after treatment of the injured and inflamed disks, which demonstrated that the anti-inflammatory HA macromolecules could tailor the disk environment to promote native IVD matrix production (Kazezian et al., 2017). Sloan et al. (2017) reported that AF and NP biomaterial repair strategies were used individually and combined to treat IVD degeneration modeled in *ex vivo* rat-tail motion segments by annulotomy and nucleotomy. A riboflavin cross-linked collagen hydrogel was injected into the defects in the AF while a modified HA hydrogel was injected for the NP repair (Figure 4A). MRI showed that simple AF and NP repair could only restore 1/3 hydration, and there was no significant difference between repair treatment and hydration (Figure 4B). Mechanical test exhibited that combined treatment of biomaterial AF and NP repair was effective at increasing NP hydration from NP repair alone (Sloan et al., 2017).

Hyaluronic acid hydrogels could also be mixed with other natural hydrogels such as gelatin or synthetic polymers like PEG. The addition of gelatin provided viscoelastic properties like native NP without hindering the NP cells regeneration. While the HA combined with PEG was found to be beneficial to the proliferation of NP and AF cells (Endres et al., 2010; Collin et al., 2011; Frith et al., 2013). Altogether, although HA is an interesting material for IVD regeneration since it is an integral part of native IVD tissues, its main setbacks are the differential effects of molecular weight, because higher molecular weight of HA hydrogels resembles the IVD mechanically and lower molecular weight formulations promote the cell function. Therefore, based on the biocompatible and biodegradable properties, the versatile biomaterial of HA is allowed for adhesion of many different cell types examined for IVD regeneration, possessing great potentials for the NP regeneration.

### Collagen and Gelatin

Collagen, as an important component of ECM, is a natural biological material and widely found in skin, bone, cartilage, blood vessels, teeth, and tendons (Ferreira et al., 2012). Gelatin is a substance extracted from animal collagen and obtained by the thermal denaturation. Since they are biodegradable with non-toxic biodegradation products, collagen and gelatin hydrogels have great applications in reconstructive surgery, drug delivery, wound healing and tissue regeneration (Huang et al., 2005; Bron et al., 2012). Collagen I hydrogels can remain good rheological behaviors to resemble native NP and collagen matrix can restore the disk height and mechanical behaviors of spinal segment (Wang et al., 2019). Gelatin, mostly derived from collagen I, is always applied by addition of other components (e. g. HA) to improve viscoelastic properties. Tsaryk et al. (2015) analyzed a specific formulation of collagen-low molecular weight of HA semi-interpenetrating network loaded with gelatin microspheres for IVD tissue engineering. After injection into defective parts, composite hydrogels could not induce the inflammation with favorable growth and chondrogenic differentiation potential of mesenchymal stem cells (MSC) and nasal chondrocytes (NC) *in vitro* and *in vivo*, promoting chondrogenic phenotype and representing the suitable candidates for NP tissue engineering (Tsaryk et al., 2015). Similarly, adding gelatin into other hydrogels not only affected mechanical properties but also improved the microenvironments of NP cells because gelatin was benefit for cell attach and function. In addition, gelatin-based hydrogels can inhibit the progression of IVD degeneration in NP-suction rabbits *in vivo*. Moreover, after injection of cell-free gelatin microspheres into a rabbit disk degeneration model, the apoptosis rate of NP group was lower than that of IVDs group without any treatments (Li et al., 2008; Chen et al., 2013).

In a word, collagen and gelatin hydrogels, as a part of ECM, are a series of biomaterials to favor the cell adhesion, growth and proliferation, but they have poor thermal stability above 37°C. In this case, these hydrogels always require other biomaterials to form composite hydrogels for improving their biological and medical applications.

### Agarose

Agarose, as a polysaccharide extracted from algae, consists of a D-galactose and a 3,6-anhydrous l-galactose monomer (Gruber et al., 2006). The agarose hydrogel can be formed with a 3D spiral structure once being mixed with water. None to mild immunological responses is *in vivo* with subchondral cartilage defects (Hunt and Grover, 2010). Agarose was combined with a synthetic electrospinning polymer to simulate IVD in compression and torsion tests, wherein agarose was used for NP and electrospinning polymer was served for AF. Bovine NP cell culture infused with transforming growth factor (TGF-β) in an agarose hydrogel had the ability to respond with cyclic compression loads and increase gene expression, because MSCs seeded on composite structures can produce ECM components for cell growth and proliferations (Tilwani et al., 2012).

Although agarose hydrogel itself may not be suitable and sufficient for the IVD regeneration, research scientists are introducing the agarose into composite scaffolds to conduct more comprehensive mechanical tests on NP-AF analogs. Therefore, it is promising as a structural component to blend with other hydrogel formulations to development of hydrogel scaffolds for the effective IVD regeneration in clinical medicine.

## Synthetic Hydrogels

### Polyethylene Glycol-Based Hydrogels

**FIGURE 5:**
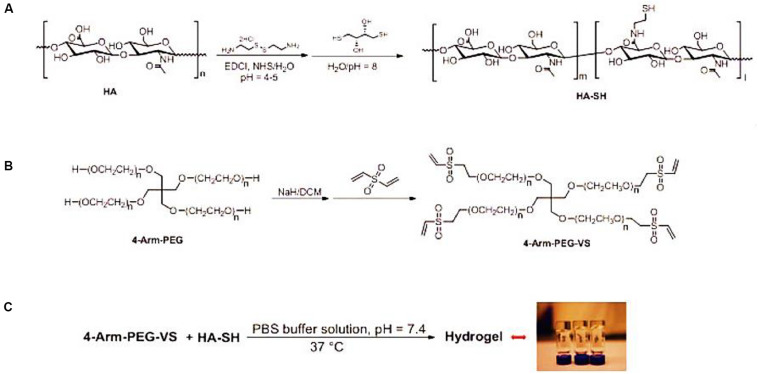
**(A)** Schematics for synthesis of HA-SH from HA, **(B)** 4-arm-PEG-VS based on 4-arm-PEG, and **(C)** formation of the HA-PEG composite hydrogel via the Michael addition reaction between HA-SH and 4-arm-PEG-VS. Reproduced from Jeong et al. (2014) with permission from Copyright 2014 Elsevier.

An important class of NP regeneration for synthetic hydrogels is based on PEG derivatives, which are hydrophilic and biocompatible polymers with the stealth-like behavior *in vivo*, i.e., they are not readily recognized by the immune system (Benoit and Anseth, 2005; Lin and Anseth, 2009; Francisco et al., 2014; Wang J. et al., 2016; Wang et al., 2016b, 2017b,c). Although PEG-based hydrogels exhibit a similar range of biomechanical properties (compression, tensile, and hydrostatic swelling) as articular cartilage, they are rarely used alone for regeneration medicine because of their non-cell adhesive behaviors. Therefore, PEG-based hydrogels are always utilized through the addition of synthetic or natural components like PLA, RGD sequences or enzyme-sensitive peptides that do allow for cell adhesion and proliferation (Alcantar et al., 2000; Lin-Gibson et al., 2004; Tessmar and Gopferich, 2007; Wang et al., 2016b, 2018b; Feng et al., 2017; Li et al., 2020b). Jeong et al. (2014) demonstrated a multiple HA-PEG composite hydrogel formulation to affect the matrix synthesis and behaviors of NP and AF cells for the IVD (Figure 5). The optimization of molecular weight and hydrogel parameters can tailor the mechanical properties (70–489 kPa), metabolite consumption, sulfated glycosaminoglycan production and multi-cell cluster morphology upon the HA-PEG hydrogels (Jeong et al., 2014).

**FIGURE 6:**
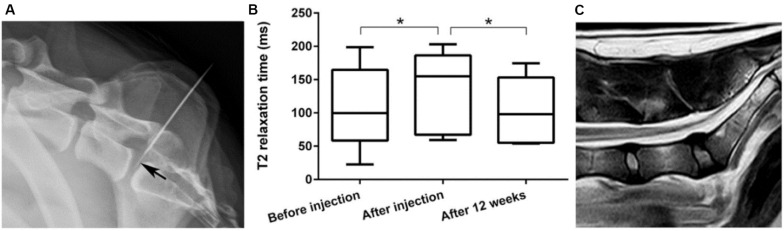
**(A)** Laterolateral radiographic images of fluoroscopy-guided intradiscal injection. Note that the 20-G needle advanced into the epidural space and the 27-G needle (arrow) positioned in the center of the NP. **(B)** T2 mapping values of the NP were significantly higher after injection compared with those at preinjection and 3 months’ follow-up. **(C)** Representative T2 magnetic resonance image 3 months after intradiscal injection with the celecoxib-loaded hydrogel. Reproduced from Tellegen et al. (2018) with permission from Copyright 2018 Wiley. *p < 0.05.

Tellegen et al. (2018) reported a poly(ε-caprolactone-co-lactide)-b-poly(ethylene glycol)-poly(ε-caprolactone-co-lactide) PCLA-PEG-PCLA hydrogel with biocompatibility and feasibility for the intradiscal application, which was evaluated in ten client-owned dogs with early spontaneous IVD degeneration by injection of celecoxib-loaded hydrogels (Figure 6). Clinical examination and owner’s questionnaire results showed that reduction of back pain of nine over ten dogs had no adverse effects, demonstrating this intradiscal injection of celecoxib-loaded hydrogels will be developed into novel treatment biomaterials and modalities for canine and human patients with chronic low back pain (Tellegen et al., 2018).

To sum up, PEG hydrogels are favorable for cell attachment and effective IVD regeneration due to their feasible eases in usage, adjustability and modification. Furthermore, PEG-based hydrogels can be considered as additions to any other hydrogel formulations for the IVD regeneration.

### Polyurethane

**FIGURE 7:**
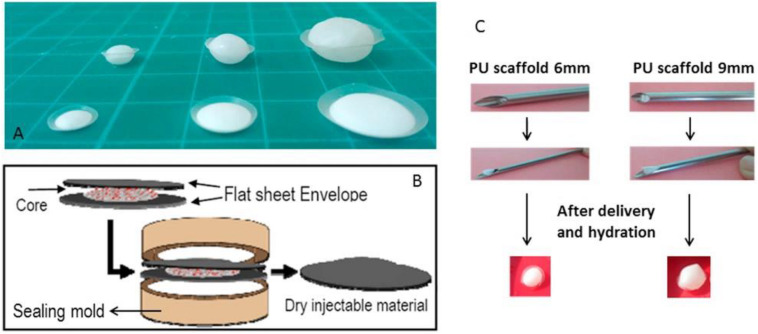
**(A)** Flat discoid shaped PU scaffold before (front) and after (back) swelling at various sizes. **(B)** Scheme of PU scaffold assembly process: core disk is wrapped by two envelope disks, and heat sealed within a custom made sealing mold. **(C)** Non-invasive delivery system for PU scaffold: a demonstration of the scaffold insertion and swelling function after delivery and hydration. Reproduced from Li et al. (2016) with permission from Copyright 2016 Elsevier.

Synthetic PU is widely used in electrical circuit housings owing to its excellent thermal stability at physiological temperatures (Santerre et al., 2005). PU and its derivatives are generally biodegradable and applied for cartilage repair for several years (Yang et al., 2009; Woods et al., 2010; Hung et al., 2014). Various PU composite scaffolds, such as PU/silk fibroin (SF) hydrogel, have been widely investigated, in which tailor of the silk/PU ratio can control the degradation rate and mechanical properties. In this case, PU/SF scaffolds was used and injected into the NP cavity to replace the NP in cadaveric porcine spines (Yeganegi et al., 2010; Hu et al., 2012; Park et al., 2013). Li et al. demonstrated a bi-phasic PU scaffold with rapid swelling property using electrospun methods. After implantation of the scaffold into a bovine whole IVD model under dynamic loading for 2 weeks, the dynamic compressive stiffness and disk height maintained good stability and scaffolds exhibited favorable cytocompatibility for native disk cells. The increased intensity of proteoglycan and type II collagen and decreased intensity of type I collagen in remaining NP tissue indicated a potential to retard degeneration and preserved the IVD cell phenotype, which may be promising for minimally invasive approaches to promote IVD regeneration (Figure 7; Li et al., 2016).

Therefore, PU-based scaffolds are suitable for IVD regeneration due to their ease of use and good biocompatibility in different forms (gels and electrospun scaffolds) for various biomedical fields, but there are few reports about the interaction between NP cells and PU scaffolds so far.

### Poly(-ε-caprolactone)

**FIGURE 8:**
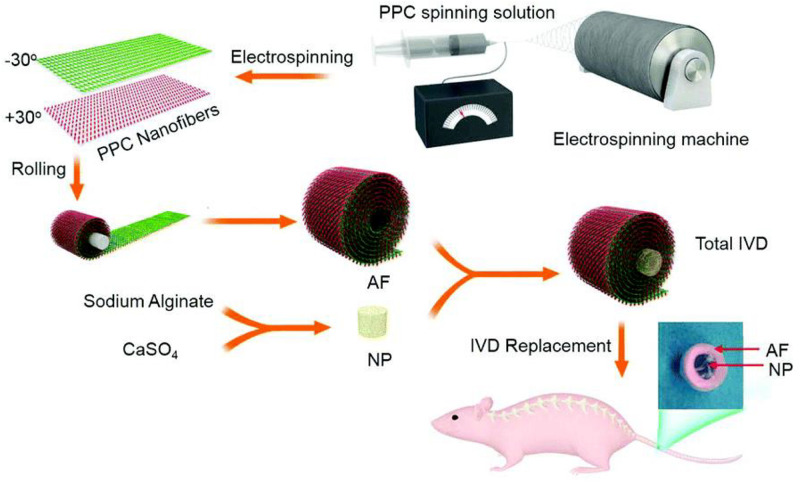
Schematic illustration of the procedure of IVD preparation and replacement using blended PCL/PLGA/type I Collagen spinning solution. Reproduced from Yang et al. (2017) with permission from Copyright 2017 Royal Society of Chemistry.

As a typical synthetic polyester, PCL is approved for usage in the human by FDA and shows great potentials in various medical applications, which can be degraded through hydrolysis of ester linkages with slow degradation time from months to years (Nerurkar et al., 2009; Guarino et al., 2010; Dash and Konkimalla, 2012). PCL scaffold is shown to be a complete IVD replacement to hold promise for IVD regeneration because electrospun PCL fibers can mimic an AF fiber structural alignment and provide high mechanical properties. In general, PCL-based scaffolds require other synthetic scaffold materials to form the injectable hydrogels, wherein the PCL plays important roles in the enhancement of strength and prolongation of service life for PCL-based hydrogel scaffolds (Lopez et al., 2010; Martin et al., 2014; Zhou et al., 2020). Yang et al. (2017) prepared a tissue engineered IVD to reduce the chronic neck and back pain, which consisted of an alginate hydrogel-based NP and concentric ring-aligned electrospun PCL/PLGA/Collagen type I-based AF. This engineered IVD possessed excellent hydrophilicity, structural and functional performances to simulate the native IVD and treat IVD degeneration after implantation for a long period *in vivo* (Figure 8; Yang et al., 2017).

Therefore, PCL is an FDA-approved material for use in the human body, possessing great promise as an AF regeneration component due to its high tensile strength to reinforce the hydrogel structures, especially for the whole IVD replacement/regeneration using the 3D printing technique for the disk-like structures.

## Summary and Perspectives

In this review, we had attempted to provide an overview of recent advances of biocompatible hydrogels for IVD regeneration. Critically, we tried to underline the attractiveness of mechanical properties, regeneration capabilities and suitability for IVD regeneration medicine. Also, we intended to give a glimpse of the physical-chemical strategies on the intelligent fabrication of naturally derived (chitosan, alginate, hyaluronan, collagen, and agarose) hydrogels and synthesized biopolymer (PEG, PU, and PCL) hydrogel scaffolds. The literatures discussed in this review are by no means complete. We only selected and highlighted some typical examples to raise the reader’s interest and awareness about the hydrogels for biomedical IVD regeneration applications.

Over the past two decades, scientists had made significant efforts to treat the IVD degeneration by development of many novel strategies and technologies. These socio-economic impacts had caused a serious annual cost problem for a lot of patients all over the world. Although considerable knowledge and current treatments have been achieved in cartilage tissue and IVD repair, human disability and pain will remain as a problem for a long time. For example, difficult assess of accurate IVD damage greatly hinders the design of most substitutes, and there is still no definition for the pathological state as the cause of degeneration up to date. In addition, high complexity and heterogeneity of IVD requires an increase in basic knowledge to fully understand all related processes and details to IVD degeneration. The feasibility of IVD regeneration strategy lies in the integration between native organizations and new functional organizations. Frequent therapy methods include biomaterials, cells, biomolecules and genes through the injection pathways. Nevertheless, so far, there is still no way to achieve long-term success using a single tissue for effective IVD regeneration. New approaches, such as combining hydrogel scaffolds with cells/biomolecules/genes, are currently being researched to repair two different tissues of AF and NP. For many engineering fields, imitating the natural environment outside the body may be a huge challenge, especially for the focuses on replicating mechanical stimulation *in vivo*.

Tissue engineering and regenerative medicine have become the most important strategies for treating diseases and repairing tissues, especially for IVD degeneration. Hydrogels are the optimal candidates to promote their developments for medical applications, because they can provide direct mechanical support and guide the differentiation of cells in IVD and the production of ECM. Wherein, naturally derived hydrogels have the advantages of good biocompatibility, safety and biodegradability, which provide a more favorable and suitable environment for cell adhesion, growth, proliferation and regeneration, but poor solubility, cost manufacturing processes and regulatory physical-chemical issues greatly hinder their wide applications. In comparison, synthetic hydrogels are feasible to adjust their chemical properties, but the by-products of degradation or residual monomer are always harmful in some cases that limits the interaction with cells and tissues. To address these problems, most of hydrogels are blended in order to possess the combined properties of biodegradability, biocompatibility, processability and mechanics, thereby favoring the IVD regeneration and tissue repair. Through high-throughput screening of biomaterials, several characteristics of hydrogels have been related to their regenerative effects, which provide more possibilities on the effective design of composite hydrogel scaffolds to satisfy the requirements of clinical treatments. For example, a fabricated photo-polymerizable poly (ethylene glycol) dimethacrylate nano-fibrillated cellulose composite hydrogel could *in situ* administer via a customized minimally invasive medical device, which restored the function and height of degenerated IVD in bovine disk model and was mechanically resistant even after half a million loading cycles compared to other hydrogels. It is clear that hydrogels with advanced capabilities display a promising future for NP replacement (Schmocker et al., 2016).

For NP repair, engineered hydrogel scaffolds need to restore the IVD height and motion segment stability, possess sufficient durability to maintain physical support, provide suitable environments for NP cells to avoid inflammation, and have abilities to protect the AF tissue. For AF repair, engineered hydrogel scaffolds need to mimic the collagen fiber architecture, which orients microstructure and high mechanics like the native AF. However, this may involve a considerable development lag time for NP and AF regeneration materials. In addition, since the clinical application of hydrogels may require surgical techniques for further research and large diameter of needle injections with amounts of liquid injections to accelerate the IVD deterioration, smart hydrogel is preferably injectable with small size of needle for NP regeneration. Unfortunately, although the application of biomaterials for regeneration of the IVD appears to achieve the enhanced progress in this area, only one hydrogel has made it into a clinical trial until now. Therefore, it is necessary to establish a scientific community with the lessons learnt from the literature through this review and fabricate a methodology or criteria on the design and development of final biological IVD products for regenerative medicine, which makes the hydrogel scaffolds satisfy more advantages on adjustable structure, better strength, adequate immune response and good biodegradability for enabling the real applications in human patients.

## Author Contributions

XW, CL, and XY initiated the project. GT, BZ, FL, WW, XW, and YL searched the data base, wrote and finalized the manuscript. All authors contributed to the article and approved the submitted version.

## Conflict of Interest

The authors declare that the research was conducted in the absence of any commercial or financial relationships that could be construed as a potential conflict of interest.
